# Structural Unfolding of G-Quadruplexes: From Small Molecules to Antisense Strategies

**DOI:** 10.3390/molecules29153488

**Published:** 2024-07-25

**Authors:** Giorgia Fracchioni, Sabrina Vailati, Marta Grazioli, Valentina Pirota

**Affiliations:** 1 Department of Chemistry, University of Pavia, via Taramelli 10, 27100 Pavia, Italy; giorgia.fracchioni01@universitadipavia.it (G.F.); sabrina.vailati01@universitadipavia.it (S.V.); marta.grazioli01@universitadipavia.it (M.G.); 2 G4-INTERACT Group, Universal Scientific Education and Research Network (USERN), 27100 Pavia, Italy; 3 PhD National Program in One Health Approaches to Infectious Diseases and Life Science Research, Department of Public Health, Experimental and Forensic Medicine, University of Pavia, 27100 Pavia, Italy

**Keywords:** G-quadruplex, disrupting small molecules, antisense strategy, G-clamp, locked nucleic acid, new therapeutic strategies

## Abstract

G-quadruplexes (G4s) are non-canonical nucleic acid secondary structures that have gathered significant interest in medicinal chemistry over the past two decades due to their unique structural features and potential roles in a variety of biological processes and disorders. Traditionally, research efforts have focused on stabilizing G4s, while in recent years, the attention has progressively shifted to G4 destabilization, unveiling new therapeutic perspectives. This review provides an in-depth overview of recent advances in the development of small molecules, starting with the controversial role of TMPyP4. Moreover, we described effective metal complexes in addition to G4-disrupting small molecules as well as good G4 stabilizing ligands that can destabilize G4s in response to external stimuli. Finally, we presented antisense strategies as a promising approach for destabilizing G4s, with a particular focus on 2′-OMe antisense oligonucleotide, peptide nucleic acid, and locked nucleic acid. Overall, this review emphasizes the importance of understanding G4 dynamics as well as ongoing efforts to develop selective G4-unfolding strategies that can modulate their biological function and therapeutic potential.

## 1. Introduction

The Watson and Crick double helix, or DNA-B, is one of the most fascinating structures due to its fundamental role in biology. It is the key to deciphering the genetic code, with genetic information encoded in the pairing of nucleotide bases [1]. For decades, the scientific community has considered this canonical B-form as the only relevant structure; however, during biological processes, nucleic acid sequences can adopt transient conformations. This phenomenon, known as structural polymorphism, is influenced by various factors such as the oligonucleotide sequence; solution conditions; hydration; the presence of ions, proteins, or ligands; and superhelical stress [2]. Non-B structures include G-quadruplexes, Z-DNA, parallel triplexes, i-Motifs, cruciforms such as Holliday and Three-way junctions, A-motifs, and slipped-strand structures [3].

**Figure 1:**
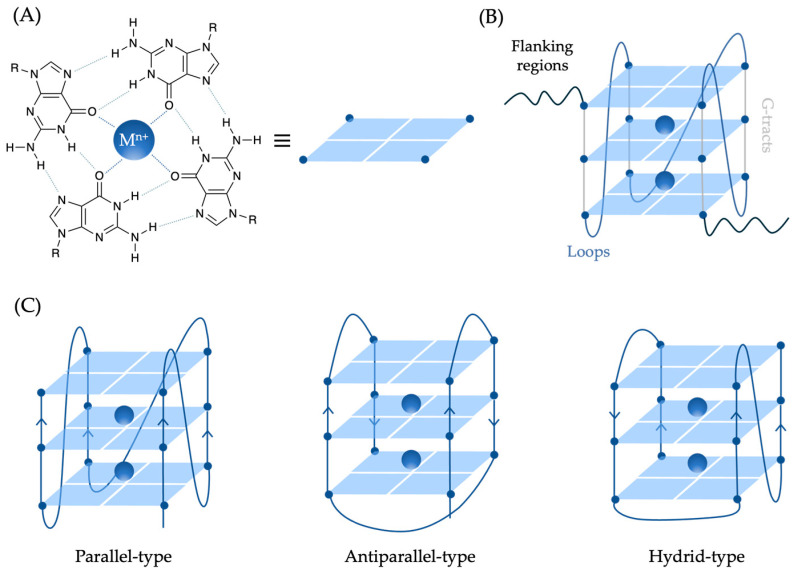
Schematic representation of G-quadruplex structures. (**A**) Square-planar G-quartet; (**B**) backbone of the intramolecular G4 structures, common to every G4s, with loops and flanking regions differentiating structural elements; and (**C**) parallel-, antiparallel-, and hybrid-type topologies of G4 structures.

Over the past two decades, G-quadruplexes (G4s) have gathered significant interest in medicinal chemistry due to their unique structural properties and potential roles in various biological processes and diseases [4]. Under physiological conditions, four guanine bases can interact via Hoogsteen-type hydrogen bonds to generate square-planar guanine quartets, known as G-tetrads. Each guanine base acts as a hydrogen bond donor and acceptor with its neighbors, involving the N1, N7, O6, and N2 atoms (Figure 1A) [5]. Two or more G-tetrads can stack on top of each other, constituting the backbone of the G4 structures common to all G4s. These highly polymorphic structures are stabilized by both π-π interactions and monovalent cations, like K^+^ or Na^+^, which are centrally coordinated to the O6 atoms of the guanines and positioned between successive tetrads (Figure 1A,B) [6]. The structural differentiating elements of G4s are (i) the grooves formed by stacking tetrads, which can vary in width and depth depending on the nucleotide sequence; (ii) loops corresponding to the nucleotide sequences that connect the guanine strands within the G4; and (iii) flanking regions, which are additional nucleotides at the 5′ and 3′ ends that are not part of the G4 stem but can influence its stability and conformation (Figure 1B). Additionally, depending on the orientation of the G-tracts, three different G4 topologies are possible: parallel if all strands have the same orientation; antiparallel if two filaments point in the same direction and the other two in the opposite one; and hybrid if three strands have the same direction and the fourth is oriented oppositely (Figure 1C) [7]. Moreover, G4 structures can be intramolecular, originating from a single nucleotide sequence, or bimolecular or tetramolecular, involving the intermolecular interaction of two or four nucleotide filaments, respectively [8].

G4s are not randomly distributed but occur in key guanine-base-rich genome regions, such as telomeres, gene promoters, and untranslated regions (UTRs) [6,9,10,11]. Over the years, growing evidence has revealed that G4s behave as pivotal structural elements in regulating different biological processes. For instance, G4s within gene promoters influence the binding of transcription factors and the RNA polymerase, modulating gene expression by either boosting or inhibiting it [11,12]. G4 structures in the 5′-UTR of mRNAs can affect translation initiation [13,14], while telomeric G4s are recognized as crucial motives for regulating telomerase activity and protecting chromosome ends [15,16]. They can influence the epigenetic landscape, which in turn affects chromatin structure and DNA accessibility to transcriptional machinery [17,18]. Moreover, G4s can play a role in the regulation of DNA replication [19,20] and maintain genome stability by regulating homologous recombination and DNA repair processes [21,22].

For these reasons, their selective folding modulation is emerging as a pivotal advance in drug discovery for developing novel therapeutic strategies against cancer [23,24], viral infections [25,26], parasite diseases [27,28,29], bacterial infections [30,31,32], and, more recently, neurodegenerative diseases [33,34].

Ongoing research has primarily focused on enhancing the selectivity and potency of G4-stabilizing ligands, with some compounds progressing to preclinical and early clinical trials [35,36]. The goal is to enable these ligands to specifically recognize and stabilize G4s over other nucleic acid structures [37,38,39] or, even more challengingly, to distinguish and interact with a single G4 structure among many [26,40].

The rationale behind this focus stems from the conventional understanding that G4 stabilization prevents the molecular recognition of the target sequence by altering its geometry. In the first instance, this is the case of anticancer strategies targeting telomerase, which is frequently upregulated in cancer cells. The stabilization of telomeric G4s by small molecules prevents telomerase from binding and elongating telomeres, crucial for cancer cell immortality [41,42,43]. In parallel, it was postulated that stabilizing G4 structures within gene promoters acts as a physical barrier, hindering transcription factor recruitment and/or stalling polymerase progression, ultimately leading to gene inactivation [9]. In this regard, stabilizing the folding of G4 structures within oncogene promoters is favorable to cancer treatment [23], whilst disrupting them is beneficial in the context of neurodegenerative disorders. In fact, G4s have been identified as pathogenic factors in neurodegenerative disorders since, for example, they can sequester proteins involved in splicing regulation; inhibit the transcription process of genes involved in synaptic function, neuronal growth, and survival; promote R-loop formation and RNA polymerase stalling; and influence the expression and aggregation propensity of neuronal proteins exacerbating toxicity in neurons [33].

Interestingly, the development of G4-selective antibodies [44] has enabled genome-wide mapping of G4s through Chromatin Immunoprecipitation followed by sequencing, allowing for a more direct investigation [45]. These studies revealed an almost inverted picture to the earlier investigations; G4 structures are predominantly enriched at gene promoters of actively transcribed genes [46,47]. This suggests, contrary to the previous assumption, that G4s might function as positive regulators of gene expression by enhancing the transcriptional activity rather than repressing it [9]. As a result, from a medicinal chemistry standpoint, developing selective tools directed at destroying G4 structures rather than stabilizing them could be a potential technique for repressing transcriptional and/or translational pathways, also in the case of G4s within oncogene promoters. This strategy could serve as the foundation for developing tailored medicines that target specific genetic and molecular pathways involved in a variety of disorders.

Supporting this approach is the knowledge that higher-order DNA and RNA G4 structures are biologically resolved by helicases. Helicases are DNA- and RNA-unwinding enzymes, discovered in 1976 [48,49], which play a crucial role in genome maintenance [50], DNA damage response, DNA repair [51], and ribosome biogenesis [52]. To date, 95 different helicases have been reported, highlighting their diverse functions and significance in cellular processes. The dysregulation of helicases has been linked to genetic disorders, cancer predisposition, and neurodegenerative diseases [53], emphasizing the importance of helicases in maintaining genomic integrity and the potential consequences of their malfunction. Given the critical role of helicases in resolving G4 structures and the impact of their dysregulation, the design and synthesis of helicase surrogates, which mimic this activity, present a promising therapeutic strategy [54]. By mimicking the natural activity of helicases, these surrogates could potentially restore normal cellular function through G4 flattening, counteracting the effects of helicase dysfunction.

Given these new perspectives, there is a growing interest in developing synthetic strategies that can selectively modulate G4 folding. Therefore, in parallel with the engineering of more selective G4-stabilizing ligands, the design of effective new strategies aimed at unfolding G4s has recently gained traction to shed new light on the complex roles of G4s in gene regulation and disease pathology.

This review provides a comprehensive overview of the latest synthetic strategies aimed at disrupting G4 structures, emphasizing both small molecules and antisense technologies.

## 2. G4-Unfolding Small Molecules

Small molecules designed to interact with G4s potentially offer several advantages for the effective disruption of these structures, providing promising avenues for the development of novel therapeutic strategies. One of the primary advantages resides in the well-defined G4 structures with distinct features, such as the planar arrangement of guanine tetrads and unique loop configurations, which can be selectively recognized by small molecules using a targeted strategy analogous to drug targeting of folded proteins but at a nucleic acid level [9]. This has the potential for high specificity and selectivity, lowering the likelihood of off-target interactions and thereby minimizing possible toxicity and side effects. Additionally, small molecules are typically easier to administer and develop than other therapeutic modalities such as nucleic acid-based therapies or protein-based interventions [55]. They can be designed for oral, intravenous, or topical administration, providing flexibility in treatment options. They are generally stable and rarely need specialized storage conditions, making them attractive to the biopharma community. Moreover, the chemical synthesis and optimization of small molecules are well-established processes, facilitating the rapid development and scalability of potential therapeutics [56].

To date, only a very limited number of small molecules capable of effectively destabilizing G4 structures have been reported. As highlighted above, this lies in the long-standing belief that the best way to use G4s as possible therapeutic targets was to achieve their selective and strong stabilization, rather than destabilization. Once the scientific community realized the ever-increasing need to study the modulation of G4 folding to better understand their roles in various biological contexts, the main obstacle in the field remained the lack of reliable and standardized protocols that delineate consistently efficient G4 destabilization effects. This problem has been highlighted several times in the literature, demonstrating the variability of experimental results based on different methodologies [57]. Indeed, G4 ligand interactions are influenced by several factors, including ionic conditions, ligand concentrations, and the specific sequence and topology of G4s. A striking example is demonstrated by the well-known G4 binder TMPyP4 (see Section 2.1). So, the need for standardized methods is crucial to ensure the reproducibility and comparability of results across various research groups and guarantee valuable mechanistic insights into G4 biology.

In this chapter, we report examples of small molecules capable of unfolding G4 reported in the recent literature, highlighting the experimental settings and methodologies used to enable critical analysis of the data.

### 2.1. TMPyP4: A Controversial Story

**Figure 2:**
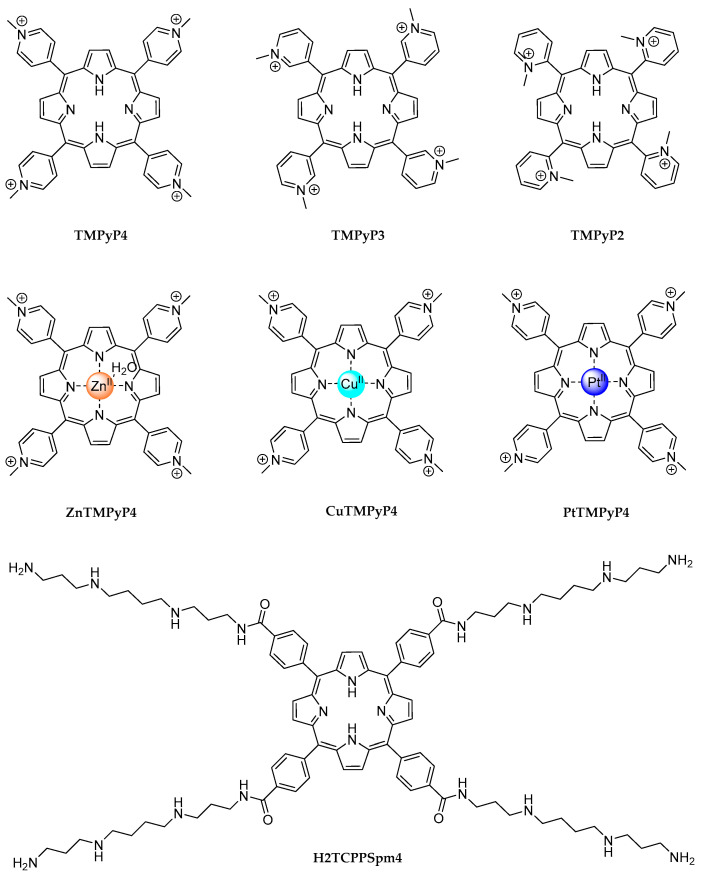
Chemical structures of TMPyP4; its positional isomers TMPyP2 and TMPyP3; its metal-complexes ZnTMPyP4, CuTMPyP4, or PtTMPyP4; and H_2_TCPPSpm4 derivative.

The first identified ligand capable of unwinding G4 structures was 5,10,15,20-tetra(N-methyl-4-pyridyl)porphin, commonly known as TMPyP4 (Figure 2). It is a porphyrin-based compound extensively utilized, over the years, for stabilizing G4 structures despite its relatively poor selectivity for G4s over other DNA structures [37]. Its planar aromatic structure allows it to effectively overlap with external G-quartets, due to similarity in size and geometry, engaging in strong π-stacking interactions. Additionally, its positively charged pyridinium arms electrostatically interact with the negatively charged phosphate groups of the G4 grooves or loops. Altogether, these features enhance TMPyP4’s binding affinity, contributing to its stabilizing effects. TMPyP4’s interaction with telomeric DNA G4s has shown promising results in inhibiting telomerase activity in various cancer cell lines [58,59,60], and its binding to G4s within promoter regions has been reported to downregulate the expression of several oncogenes [37].

On the other hand, the first evidence of the G4-unfolding activity of TMPyP4 was reported by Fry’s research group [61]. Their work demonstrated the ability of TMPyP4 to destabilize a model DNA G4 oligonucleotide that folds from (CGG)_n_ trinucleotide repeats involved in Fragile X syndrome. Fragile X syndrome is the most prevalent hereditary cause of mental disability, whose principal hallmark is the dynamic expansion of more than 55–2000 (CGG)_n_ trinucleotide repeats, which are located in the FMR1 gene’s first exon (d(CGG)_n_) and the 5′-UTR of the FMR1 mRNAs (r(CGG)_n_). These repeated triplets spontaneously adopt hairpin structures, which further fold into intra- and intermolecular G4s, thus contributing to the transcriptional and translational silencing of FMR1 [62]. Therefore, an attractive therapeutical approach might be the destabilization of the G4 structures folded in the expanded d(CGG)_n_ sequence. Thermal stability assays (conducted in 20 mM Tris-HCl buffer, pH 8.0, with 1.0 mM dithiothreitol, 0.5 mM EDTA, 20 mM KCl, and 20% glycerol) demonstrated that TMPyP4 destabilized G′2 3′-tail d(CGG)_7_ G4 model-oligonucleotide (5′-d[(CGG)_7_CGTGGACTG]-3′) but not the telomeric DNA-G4 G′2 TeR2 (5′-d[TAGACATG(TTAGGG)_2_TTA]-3′) [61]. Indeed, in the absence of TMPyP4, the melting temperature (T_m_) of 20 nM G’2 TeR2 DNA was 44.6 ± 2.0 °C, increased by approximately +13 °C in the presence of 14 equivalents of TMPyP4 (0.3 μM, T_m_ = 57.8 ± 0.3 °C). Conversely, 0.3 μM TMPyP4 caused a notable decrease of nearly −15 °C in the T_m_ of 10 nM G’2 3′-tail d(CGG)_7_, from 45.8 ± 0.7 °C to 30.9 ± 1.0 °C [61]. TMPyP4’s destabilizing effect on tetraplex DNA was not limited to G′2 3′-tail d(CGG)_7_. It also effectively disrupted G’2 forms of non-tailed d(CGG)_7_ (5′-d(CGG)_7_-3′), 5′-tailed d(CGG)_7_ (5′-d[GTCAGGTGC(CGG)_7_)]-3′), and the hypermethylated d(5-meCGG)_7_ oligomer (5′-d(5-meCGG)_7_-3′), where each of its seven cytosine residues is 5-methylated [61].

In a subsequent study, the same research group highlighted the potential of TMPyP4 in also unfolding RNA-G4s derived from r(CGG)_n_ repeats in vitro [63]. Specifically, they previously demonstrated the ability of a [5′-^32^P]-labeled RNA transcript of pCS107(CGG)_33_ to fold into intramolecular G4 in the presence of 10 mM KCl [64]. Furthermore, they emphasized that the expression of the G4-disrupting proteins hnRNP A2 or CBF-A in HEK293 cells significantly restored the translation block induced by a (CGG)_99_ sequence positioned upstream of a firefly luciferase (FL) reporter gene [64]. Motivated by these findings and aiming to identify a clinically applicable therapeutic drug, they tested the efficacy of TMPyP4. Electrophoretic mobility analysis demonstrated that TMPyP4 (5.0 mM) could affect the folding of ^32^P-labeled pCS107(CGG)_33_ RNA transcript into a G4 structure in the presence of 20 mM KCl, suggesting its destabilizing ability [63]. Concurrently, TMPyP4 (from 50 to 350 nM concentration) enhanced the translation of 5′-(CGG)_99_-FL mRNA transcripts (pT7FMR1-5_0_-UTR(CGG)_99_-FL) in a rabbit reticulocyte lysate by up to 3-fold. At the same time, it did not alter the in vivo translation efficiency of (CGG)_30_-FL mRNA, which represents the typical number of triplet repeats in the FMR1 gene in the normal human population [64]. In addition, the combination of high relative levels of TMPyP4 (20 µM) and CBF-A G4-unfolding protein (2.9 µg of pCMV2-Flag CBF-A plasmid) induced an additive enhancement of 5.2-fold of the translation efficiency of (CGG)_99_-FL mRNA (50 ng pFMR1-5_0_-UTR(CGG)_99_-FL reporter plasmids) in HEK293 cells (compared to an enhancement of 2.8-fold in the presence of TMPyP4 alone and 3.2-fold by CBF-A alone). A synergistic effect was observed by using low relative amounts of TMPyP4 (20 µM) and CBF-A (2.5 µg) versus pFMR1-5_0_-UTR(CGG)_99_-FL reporter plasmids (500 ng), with an FL translational efficiency of 3.8-fold (compared to an enhancement of 0.8-fold with TMPyP4 alone and 1.0-fold with CBF-A alone) [64].

Interestingly, positional isomers of TMPyP4, specifically the cation porphyrins TMPyP2 and TMPyP3 (Figure 2), were initially reported to be ineffective at unfolding quadruplex structures [63]. Nevertheless, subsequent biophysical studies demonstrated that 2.4 equivalents of TMPyP3 (4.8 μM) were sufficient to effectively unfold the telomeric antiparallel G4 d(TTAGGG)_4_ (2 μM) in a high-sodium concentration environment (0.1 M NaCl and 10 mM phosphate buffer) [65].

The disrupting properties of TMPyP3 were also demonstrated by Kukreti and colleagues in two subsequent studies [66,67]. First, they reported the recognition and destabilization of a parallel G4 adopted by a 23-mer G-rich sequence, HM23 (5′-TGGGTGCCGTTGGGGGTGGGGGT-3′), located in the promoter region of the Human Myosin Heavy Chain b gene. Circular dichroism (CD) melting studies (in 100 mM KCl and 20 mM sodium cacodylate buffer pH 7.4) highlight a ΔT_m_ of −34 °C for TMPyP4 and of −17 °C for TMPyP3 at a 1:4 HM23 (3 μM):phorphyrin (12 μM) ratio [66]. Two years later, the same researchers demonstrated the unfolding of another parallel G-quadruplex, formed by a 33-mer regulatory sequence, TP (5′-CGGGCGCCGGGGCGGGGCGGGGTGGGGCGGGGC-3′), present in the promoter region of the multidrug resistance protein1 transporter gene. Titrating increasing concentrations of TMPyP4 and TMPyP3 ligands (in 100 mM KCl, 0.1 mM EDTA, and 20 mM sodium cacodylate buffer pH 7.0) resulted in a decrease in the intrinsic CD signals of TP at 262 and 245 nm, reflecting the disappearance of the quadruplex structure. The unfolding was confirmed by UV-thermal denaturation, which showed a considerable decrease in the melting temperature of the quadruplex with both TMPyP4 (ΔTm = −23.5 °C) and TMPyP3 (ΔT_m_ = −15.5 °C). Both works underline that, besides TMPyP4, TMPyP3 can also destabilize G4 structures. Nevertheless, TMPyP4 exhibits better destabilizing properties. This could be attributed to the different positions of the N-methyl groups on the pyridyl ring. In TMPyP4, the *para* position of the N-methyl group allows the four meso-pyridyl rings to rotate and easily align coplanar with the porphyrin chromophore; by contrast, the bulky N-methyl groups in TMPyP3 are *meta*-positioned, and pyridyl ring rotation is, to some extent, sterically hindered [67].

TMPyP4 was also found to unfold the thrombin-binding aptamer G4 (d(GGTTGGTGTGGTTGG)), interfering with its active conformation, and acting as an antagonist in the aptamer-mediated inhibition of blood clotting in a concentration-dependent manner. CD studies reveal that in the presence of TMPyP4 at a 1:4 G4:ligand ratio (5 μM and 20 μM, respectively, in PBS, pH 7.4), the characteristic positive band at 295 nm of antiparallel G4 decreases in intensity by over 80% [68].

The G4-unfolding ability of TMPyP4 was also reported by Basu and colleagues, who verified the destabilization of an unusually stable intramolecular RNA G4, M3Q, formed by a 20-nucleotide all-purine sequence located in the 5′-UTR of the MT3-MMP mRNA (r [5′-GAGGGAGGGAGGGAGAGGGA-3′]) [69]. The decrease in the positive CD signal at 263 nm, characteristic of a parallel G4, was observed at a 1:25 G4:ligand ratio (4 μM and 100 μM, respectively, in 100 mM KCl), indicating the unfolding of the M3Q-G4 structure. This result was also confirmed by ^1^H NMR spectroscopy; when pre-folded M3Q G4 (0.42 mM, in 20 mM potassium phosphate, pH 7.0, and 8% D_2_O) was titrated with 0.2 equivalent increments of 55 mM TMPyP4, a decrease in the intensity of the peaks of the imino protons involved in Hoogsten hydrogen bonding was observed until a complete absence of signals [69].

More recently, Pirota et al. described the TMPyP4 ability to destabilize a new G4 structure, pSNCA, actively involved in the regulation of SNCA gene transcription [34]. By CD melting experiments, a destabilization from 2 to 11 °C was recorded for two different model oligonucleotides (2.5 µM pSNCA or pSNCAext, an extended sequence comprising the three previous bases and the three subsequent ones) after the addition of four equivalents of TMPyP4 in a 10 mM lithium cacodylate buffer, pH 7.4, with 1 mM KCl and 99 mM LiCl [34].

In addition, it should be noted that the presence and type of metal within the porphyrin core can influence the ability of TMPyP4 to either stabilize or destabilize the G4 structure. This was highlighted by measuring the melting temperature of d(TAGGG)_2_ G4 model oligonucleotide (4.2 µM) by CD in 50 mM KCl, 10 mM KPi buffer, pH 7.0, in both the absence and presence of 1 equivalent of TMPyP4, ZnTMPyP4, CuTMPyP4, or PtTMPyP4 (Figure 2) [70]. TMPyP4’s distinct metal centers produced varying G4 stabilizing properties; ZnTMPyP4 exhibits effective stabilization with ΔT_1/2_ = 13.5 °C, whilst CuTMPyP4 had a lesser effect with ΔT_1/2_ = 4.7 °C. On the other hand, PtTMPyP4 exhibited a slight destabilizing effect (ΔT_1/2_ = −2.9 °C), even on d(TAGGG)_2_ telomeric G4, over which TMPyP4 has a known stabilizing impact (ΔT_1/2_ = 13.8 °C) [70].

More recently, D’Urso et al. evaluated the unwinding properties of a different porphyrin derivative, H_2_TCPPSpm4 (Figure 2), bearing spermine pendants in the four meso positions. Their study underscored the significant influence that the protocols and techniques employed have on the outcomes of porphyrin/G4 interaction studies. Specifically, they found that the method used to attain the relative stoichiometry (titration or single addition) determined whether the parallel G4 formed by the DNA aptamer TGGAG (Hotoda sequence) was stabilized or destabilized by H_2_TCPPSpm4. When added at a 1:1 ratio, either by single addition or by titration, H_2_TCPPSpm4 forms a 1:1 end-stacking complex with the G4, resulting in its destabilization by approximately 30 °C. The addition of a second equivalent still destabilizes the structure. By contrast, the addition of two equivalents in a single portion suggests a stabilization of the G-quadruplex (ΔT_1/2_ = 24 °C) in the presence of a higher concentration of the ligand [71].

This underscores, once again, that the primary challenge in identifying effective ligands for G4 destabilization lies in the absence of reliable, standardized protocols that consistently define their interaction characteristics.

### 2.2. Effective Metal Complexes with G4-Disrupting Activity

**Figure 3:**
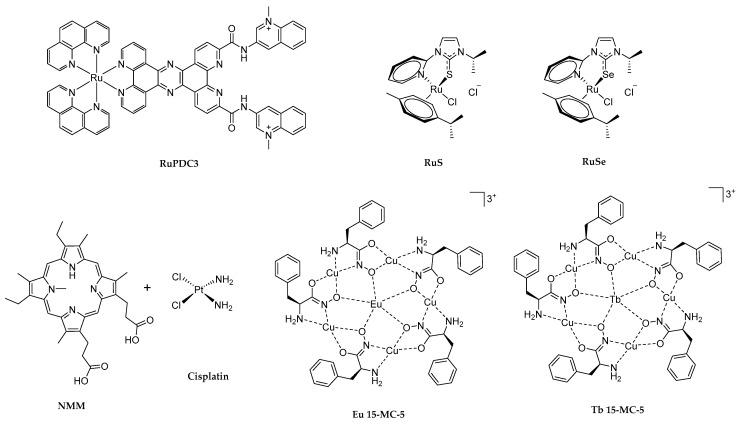
Chemical structures of the Ru(II) complexes RuPDC3, RuS, and RuSe, and of NMM-Cisplatin combination, Eu 15-MC-5, and Tb 15-MC-5.

Complexes of Ru(II) have been widely studied as DNA binders. They have interesting applications in imaging techniques and G4 visualization in live cells thanks to their large Stokes shift, environment-sensitive emission, and long-lived phosphorescent lifetimes [72,73,74]. Among all, the conjugation of a Phen-DC_3_ with a Ru(II)-tpphz moiety gave rise to Ru-PDC3 (Figure 3), a theranostic probe, water-soluble and permeable to live cells, able to detect and disrupt G4 structures [75]. Phen-DC_3_ is recognized as a high-profile G4 ligand in terms of selectivity and binding affinity [76], while tpphz was exploited due to its ability to activate the characteristic water light-switch effect when coordinated to Ru(II) complexes on DNA binding. By luminescent titration curves, the authors highlighted a clear discrimination of the Ru-PDC3 probe (10 µM in KPi buffer) for three G4s over double- (ds-) and single- (ss-) stranded DNA. They calculated one order of magnitude higher binding affinities equal to (1.4 ± 0.1) × 10^7^, (1.4 ± 0.2) × 10^7^, and (2.2 ± 0.7) × 10^7^ M^−1^, respectively for K-ras (AGGGCGGTGTGGGATAGGGAA), INTER G4 (TAGGGTTA), and 22AG (AGGGTTAGGGTTAGGGTTAGGG) G-quadruplex structures, and values ranging from 1 to 2 × 10^6^ M^−1^ for ss- and ds-DNA [75]. This affinity trend is also confirmed by analyzing the light switch effect caused by the interaction of Ru-PDC3 with all the DNA structures by fluorescence lifetime imaging. Moreover, the effectiveness of Ru-PDC3 in unfolding specific G4 structures, with varying degrees of impact depending on the G4 topology, was underscored by CD titrations (5 µM oligonucleotides in KPi buffer and Ru-PDC3 from 0 to 10 mol equiv). Specifically, the positive CD signal at 295 nm of TBA (GGTTGGTGTGGTTGG) was reduced by 82% with fiveequivalents of Ru-PDC3; the positive bands at 265 nm of Pu24T (TGAGGGTGGTGAGGGTGGGGAAGG) and K-ras decreased by 71% and 73%, respectively, with five equivalents of Ru-PDC3, while INTER G4 recorded an ellipticity decrease at 210 nm of 95% with ten equivalents of Ru-PDC3, showing the most extensive disruption. In the case of 22AG, instead, Ru-PDC3 induced a topology change from a mixed hybrid quadruplex to an antiparallel one, without any unfolding effect [75].

Different Ru(II)-based G4 unfolding ligands were reported to efficiently destabilize the intermolecular parallel d(CGG)_15_ G4, associated with neurodegenerative and neuromuscular diseases [77]. By titrating 20 µM d(CGG)_15_ with increasing amounts (from 50 to 500 equivalents) of RuS ([Ru(p-cymene)(ipit)(Cl)](Cl), or RuSe [Ru(p-cymene)(ipis)(Cl)](Cl) (Figure 3) at 25 °C in Tris-HCl buffer with 1 mM KCl, an ellipticity decrease of around 70% were recorded. This selective d(CGG)_15_ G4 disruption was also confirmed by an electrophoretic mobility shift assay and NMR titration experiments. Altogether, these results confirmed the potential therapeutic application of RuS and RuSe for neurodegenerative, neuromuscular, and neuronal disorders caused by the aberrant CGG repeats expansion [77].

In a different approach, Yang et al. developed a novel and straightforward method for highly sensitive Cisplatin (Figure 3) detection, exploiting its ability in binding and unfolding G4s [78]. Cisplatin, approved by the FDA in 1978 to treat solid tumors, has been noted for its toxic side effects, prompting the investigation of various detection methods. The authors combined the fluorescent turn-on effect resulting from the interaction between the folded PS2.M (GTGGGTAGGGCGGGTTGG) G4 and NMM (N-methyl mesoporphyrin IX), a well-known light-probe and stabilizing G4 ligand [79] (Figure 3), with the capability of Cisplatin to disaggregate the G4 structure, leading to a hypochromic effect. Fluorometric studies were conducted using solutions containing 125 nM PS2.M in 10 mM Tris–HCl buffer (pH 7.6) with 2 mM KCl and 1.5 µM NMM. The addition of increasing concentrations of Cisplatin, ranging from 1 µM to 100 µM, was monitored, highlighting a significant fluorescence emission decrease in the PS2.M/NMM complex by 41.74% after the addition of 10 µM Cisplatin (Kd = 1.19 × 10^−5^ M) [78]. The decrease of 21.04% of the elliptical positive peak at 262 nm (recorded by CD after the addition of 20 equivalents of Cisplatin) and electrophoresis studies also confirmed the interaction between Cisplatin and PS2.M with G4-unfolding-induced effects. By fluorometric data, the authors created a reliable calibration curve for Cisplatin determination with a sensitivity of 720 nM [78]. The high sensitivity and selectivity of this new method were verified by evaluating the concentration of Cisplatin in the urine samples collected from rats. In addition, this method could potentially be a useful tool for probing Cisplatin response in live-cell imaging. Indeed, this possibility was confirmed by a significant decrease in fluorescence intensity in the cytoplasm of MCF-7 tumor cells transfected with 500 nM DNA G4 and 6 µM NMM in the presence of 2 mM K^+^ after 12 h and the addition of 10 µM Cisplatin. Even weaker fluorescence was seen with 20 µM Cisplatin [78].

In the exploration of metal complexes as G4 ligands, Juskowiak and colleagues examined two 15-metallacrown–5 lanthanide complexes: Eu 15-[MCCu,pheHA]-5 (Eu 15-MC-5) and Tb 15-[MCCu,pheHA]-5 (Tb 15-MC-5) (Figure 3) [80]. Both of these two complexes were found to destabilize the human telomeric DNA-G4 dAGGG(TTAGGG)_3_ (22Htel). Each metallacrown (MC) features a ring formed by optically pure S-phenylalanine hydroxamic acid (pheHA) and copper(II) ions, with the lanthanide ion (Eu(III) or Tb(III)) in the central cavity. While this study marks the first investigation into the affinity of 15-metallacrown–5 lanthanide complexes for DNA-G4, previous research has established that MCs with the planar ring can efficiently interact with G-tetrad. The unfolding potential of the two complexes toward 22Htel was evaluated by CD spectroscopy, thermal melting experiments, and luminescence quenching of the Tb(III)–G4 system. CD spectra of 22Htel (2 µM), titrated with Eu 15-MC-5 and Tb 15-MC-5 in 10 mM cacodylate buffer, pH 7.2, and 100 mM NaCl, showed a linear decrease in the CD signal at 260 and 290 nm with increasing MC concentrations (0 µM, 4.5 μM, 13.4 μM and 19.6 μM). No new bands were detected, indicating that the G4 structure was maintained, with complete unfolding observed at a G4:MC ratio of 1:10 [80]. In the presence of 5 µM MCs, the T_m_ of 22Htel (1 µM) decreased by 16 °C (from 58 ± 1 °C to 42 ± 1 °C) with Eu 15-MC-5 and by 20 °C (to 38 ± 1 °C) with Tb 15-MC-5, confirming significant destabilization. This unfolding was found to be irreversible, as the melting profiles did not revert upon cooling. A fluorescence intercalator displacement assay using thiazole orange (TO) was also conducted to analyze G4-MC interactions. This assay exploits the fluorescence enhancement of TO when it binds to G4. The TO-22Htel complex was titrated with increasing concentrations (0–10 µM) of Eu 15-MC-5 and Tb 15-MC-5. The MCs’ unfolding effect was evidenced by a decrease in fluorescence, indicating the disruption of the TO-22Htel complex. The concentrations required to reduce the fluorescence signal by 50% were 3.40 µM for Eu 15-MC-5 and 2.68 µM for Tb 15-MC-5. The minimal difference between these results suggests that the MC-G4 interaction is influenced more by the spatial arrangement of the metallacrown rather than the nature of the central lanthanide ion. Fluorescence emission was completely quenched at ligand concentrations above 9 µM [80]. Similar behavior was observed with Tb(III)–G4 luminescence quenching, a novel G4 binding assay that exploits the enhancement of Tb(III) luminescence upon G4 binding. The mechanism behind G4 destabilization remains unclear. It was hypothesized that single-stranded DNA stabilization may occur due to non-specific interactions between the MC and the negatively charged phosphate groups, though this requires further investigation [80].

### 2.3. Other G4-Destabilizing Organic Compounds

In this subchapter, other organic compounds identified as G4-disrupting agents are reported in chronological order of publication.

**Figure 4:**
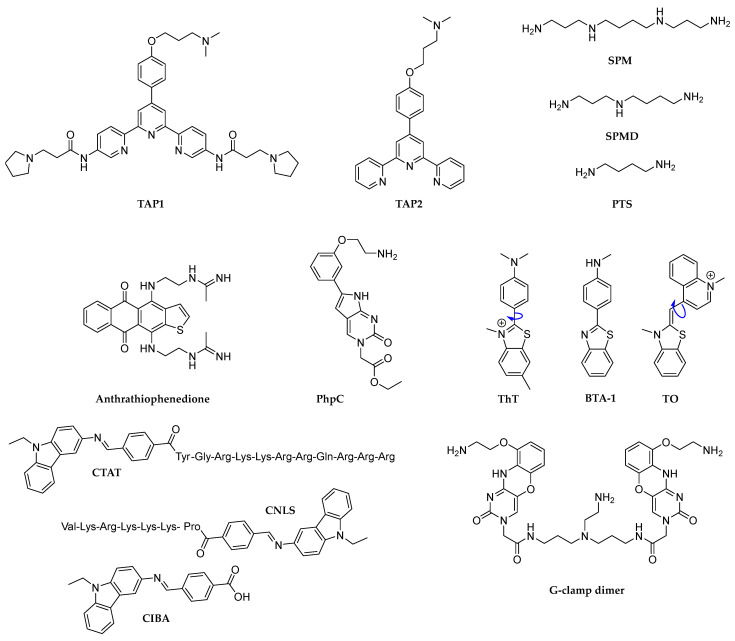
Chemical structures of the main small molecules capable of unfolding G-quadruplex structures. For the two rotamers ThT and TO the rotation is evidenced by blue arrows.

In 2009, Waller et al. described a triarylpyridine-derivative, TAP1 (Figure 4), capable of destabilizing the two G4 structures c-Kit1 (GGGAGGGCGCTGGGAGGAGGGG) and c-Kit2 (GGGCGGGCGCGAGGGAGGGG) located within the promoter of c-Kit gene [81]. The parallel dichroic spectra of 10 µM DNA-G4, in 10 mM Tris·HCl buffer at a pH of 7.4 containing 100 mM KCl, decreased in a dose-dependent manner upon the addition of TAP1 (>10 µM), indicating an apparent unfolding effect [81]. Through ^1^H-NMR spectroscopy, the authors further elucidate the ligand-induced G4-unfolding. Indeed, a decreased intensity of imino protons within 10–12 ppm [82] was recorded upon TAP1 addition, suggesting a disruption of the G-tetrads, in accordance with the CD experiments. Interestingly, this intriguing destabilizing effect is mainly due to the presence of the pyrrolidine propanamide TAP1 lateral pendants, as demonstrated by the non-significant data reported for TAP2 (Figure 4). To assess the biological behavior, the impact of TAP1 on the c-Kit gene expression was tested in HGC-27 cells, which overexpress c-Kit. A dose-dependent increase in relative c-Kit expression (compared to β-actin, which remained stably expressed) of 40%, 90%, and 150% for, respectively, 1, 5, and 10 µM TAP1 concentrations was recorded after 3 h of treatment [81]. These results indicate a correlation between the G4-disrupting ability of TAP1 and the increase in gene expression in cells, consistent with the effects observed with G4-stabilizing ligands that typically cause a decrease in gene expression.

A study by Sun et al. investigated the effects of natural polyamines on the modulation of G4-folding, revealing their potentiality in both stabilizing and destabilizing G4s depending on their concentration [83]. Polyamines, including spermine (SPM), spermidine (SPMD), and putrescine (PTS) (Figure 4), are ubiquitous cellular constituents involved in cell differentiation, proliferation, and apoptosis, suggesting that their interaction with G4s could have significant implications for tumor cells. Using CD spectroscopy, the authors elucidated dual behavior even at high potassium concentrations (150 mM). At low SPM concentrations (less than 1 mM), the telomeric H24 G4 (d(TTAGGGT), 2.5 µM) was stabilized; conversely, at higher SPM concentrations (greater than 1 mM), a destabilization effect on H24 was observed. While SPMD and PTS exhibited comparable destabilizing effects at high concentrations, they did not demonstrate significant G4-stabilizing properties at lower concentrations. This finding underscores the importance of the polyamine chain length and the number of amino groups in the stabilization of G4 structures [83]. The authors suggested that the denaturing effect may be caused by the hydrogen bonds between guanines and the polyamines’ amino or imino groups that replace H-bonding between guanines within a G4. The dual behavior was also confirmed through fluorescence resonance energy transfer (FRET), and NMR spectroscopy, which showed the impact of increasing polyamine concentrations on the instability of G4 structures. In addition, the G4-unfolding feature of SPM was also verified by CD on c-myc (d(TGAGGGTGGGTAGGGTGGGTAA)), c-Kit (d(AGGAGGGCGCTGGGAGGAGGG)) and bcl-2 (d(GGGCGCGGGAGGAATTGGGCGGG)), as well as on the two telomeric H7 (d(TTAGGGT)) and H12 (d(TTAGGG)_2_) model oligonucleotides [83].

One of the most effective classes of antitumor drugs is anthraquinone-containing antibiotics. A series of 1,4- and 2,6-difunctionalized amidoanthracene-9,10-diones have been shown to stabilize telomeric G4s, thereby inhibiting human telomerase activity [84]. Differently, Kaluzhny et al. proposed a novel anthrathiophenedione derivative (4,11-bis[(2-{[acetimido]amino}ethyl)amino]anthra [2,3-b]thiophene-5,10-dione) (Figure 4), which induced dramatic conformational perturbations on the telomeric TelQ-G4 (d(TTAGGG)_4_ in the presence of sodium ions [85]. Isothermal titration calorimetry (ITC) conducted in 10 mM sodium phosphate buffer, pH 6.5, with 100 mM NaCl or 100 mM KCl at 25 °C, revealed an association constant (K_ass_) of 10^6^ M^−1^ for a ligand–TelQNa complex and K_ass_ = (1.00 ± 0.15) × 10^7^ M^−1^ for a ligand–TelQK complex. CD titration of TelQNa at 0.8 µM with increasing ligand amounts up to 6 mM dramatically altered the CD spectra, mimicking the changes observed when TelQNa is denatured by increasing the temperature from 20 °C to 75 °C. The melting experiment indicated a partial loss of the G-quartet structure with the addition of 1 µM ligand to TelQNa, and complete quadruplex destruction at 6 µM ligand concentration at 20 °C [85]. By contrast, the compound caused only partial disorganization of TelQK, with a shift to an antiparallel G4 configuration, compared to the original spectrum of TelQK in 100 mM KCl [85].

Monchaud et al. proposed a new G-clamp87 analog, PhpC (Figure 4), to effectively induce G4-unfolding by trapping and stabilizing flipping guanine through four H-bonds [57]. In this work, they tested a panel of 14 G4 ligands to compare both stabilizing and destabilizing behaviors on a human telomeric G4 sequence. By an efficient G4-Unfold Assay (for details, please refer to reference [57]) they demonstrate that the presence of PhpC accelerates the double-strand hybridization kinetics at all working ligand concentrations (from 1 to 20 mol equiv), suggesting its potential as a destabilizer of the G4 structure. In fact, the initial hybridization velocity was found to be 1.4 to 1.8 times faster than the control (V_0_ = 51.5 s^−1^ in the absence of ligand versus V_0_ = 72.7 s^−1^ and V_0_ = 94.5 s^−1^ in the presence of 1 and 20 mol equiv of Phpc, respectively). The performance of Phpc was also evaluated by CD titrations, which showed a 17.5% drop in hTelo G4 (3 µM) ellipticity at 293 nm with 10 ligand equivalents, without discernible absorbance contributions at 257 nm, where G4 absorbs light [57]. These results were consistent with the non-denaturing polyacrylamide gel electrophoresis (PAGE) and dynamic light scattering investigations in which Phpc triggered a decrease in the hTelo-folding in a dose–response manner. Additionally, the ability of PhpC to trap a transiently flipping guanine was supported by fluorescence investigations. Titration against hTelo revealed that decreasing G4 stability by reducing potassium concentration (from 100 mM to 1 mM) led to a significant decrease in PhpC fluorescence. This decrease is attributed to the transient opening of the external G-quartet in less stable G4s, allowing PhpC to trap flipping guanines [57]. This promising unfolding behavior prompted the authors to evaluate Phpc compatibility with enzymatic processivity. Fascinating, Phpc aided the amplification of the G4 strand with a selectivity factor (defined as ΔFI_G4_/ΔFI_non-G4_) equal to 10.7 with only 1 ligand equivalent [57]. PhpC’s encouraging in vitro properties were also verified in human cells using qualitative and quantitative methods [86]. G4 landscapes were followed qualitatively by G4 imaging in live cells using the turn-on G4-selective fluorescent probe N-TASQ (50 µM, IC_50_ > 300 mM, 6 h, λ_ex_ = 405 nm; λ_em_ = 450–530 nm). PhpC (20 µM; IC_50_ > 300 µM) was preincubated on MCF7 cells to measure the quantity of N-TASQ *foci* and the intensity of fluorescence within each cell, recording a good (30%)-to-strong (68%) decrease in the G4 signal. Interestingly, the apparent affinity of Phpc for NRAS (5′-GGGAGGGGCGGGUCUGGG-3′) RNA G4 resulted in >100 µM by fluorescence quenching assay guaranteed a no-competition process with N-TASQ in G4 targeting (^app^K_d_ of N-TASQ = 0.51 µM) [86].

Q. Xu et al. observed G4 switching to a parallel-stranded duplex (psDNA) via molecular rotor clustering, using thioflavin T (ThT; Figure 4), on two human KRAS proto-oncogene sequences [87]. Specifically, the sequence 32R (5′-AGGGCGGTGTGGGAAGAGGGAAGAGGGGGAGG-3′) forms a parallel G4, whereas 32R-3n (5′-GCGGTGTGGGAAGAGGGAAGAGGGGGAGGCAG-3′), with a three-nucleotide shift, forms a unimolecular hybrid G4 at low concentrations and a dimeric antiparallel G4 at high concentrations. Upon the addition of ThT (160 µM) in 20 mM Tris–HCl (pH 7.0), containing 100 mM Li^+^, the CD spectra of both the DNA-G4s (8 µM) showed an induced CD negative signal at 445 nm, indicating psDNA formation. This switch was also observed for 32R-3n in the presence of 100 mM K^+^ but not for the parallel 32R G4 [87]. PAGE experiments confirmed that 32R-3n G4 (8 µM) can be disrupted by ThT (20 µM), while 32R G4 remained unaffected in 100 mM potassium solutions. These data were in accordance with CD-melting experiments that recorded an increase in melting temperature at 260 nm upon the addition of ThT for both KRAS G4s in lithium solution and only for 32R-3n in the potassium environment. This highlighted a G4-psDNA switching with subsequent stabilization of psDNA by the ThT rotor. A time-dependent increase in ThT fluorescence further supports this interaction, with faster psDNA formation at higher DNA concentrations (8 µM versus 1 µM). The study also compared BTA-1 (a non-rotor analog) and TO (a ThT-rotor analog cyanine dye). CD spectra and PAGE revealed that TO, like ThT, induced switching in both K^+^ and Li^+^, while BTA-1 did not induce psDNA formation [87].

P. Xu et al. prepared two peptide-carbazole conjugates, CTAT and CNLS (Figure 4), for antimicrobial activity and demonstrated their ability to unfold G-rich sequences G1 (5′-AGGGTGGGGAGGGGGG-3′) and G2 (5′-GGGCGGGCGGGAGGGAGGGG-3′) [88]. These conjugates were synthesized by coupling the carbazole derivative CIBA (Figure 4), a G4-ligand, to the N-terminus of the cell-penetrating peptide TAT (47–57: YGRKKRRQRRR) and the nuclear localization signal (NLS: PKKKRKV) peptide. Using UV-vis spectroscopy, fluorescence titration, melting experiments, and gel electrophoresis, the interaction of CTAT and CNLS with calf thymus DNA (ctDNA) was studied. The titration of ctDNA (50 µM, pH 7.4 Tris-HCl buffer solution) showed a more pronounced hyperchromic effect and a slight red shift in UV-vis spectra in the presence of CTAT and CNLS (ranging from 0 to 21 µM) indicating enhanced DNA interaction compared to CIBA, TAT, and NLS alone [88]. Fluorescence titration (50 μM ctDNA, 5 μM Ethidium bromide (EB), in 10 mM Tris-HCl buffer pH 7.4) revealed that increasing concentrations (0–21 µM) of CIBA, TAT, NLS, CTAT, and CNLS gradually quenched DNA-EB fluorescence, through a static mechanism caused by collisions between excited DNA and the drugs. Thermodynamic parameters calculated using Va not Hoff analysis indicated that hydrogen bonds and van der Waals forces were the main interactions for CIBA, TAT, CTAT, and CNLS with ctDNA, while hydrophobic interactions were dominant for the NLS-DNA system. Melting experiments in the same experimental conditions confirmed that these compounds are groove binders, not intercalators, as evidenced by the minimal changes in DNA-EB melting temperature upon their addition (5 µM). The Tm of ctDNA-EB was 88.2 °C, with minor variations upon adding CIBA, TAT, CTAT, NLS, and CNLS, supporting groove binding mode. Gel electrophoresis further confirmed this interaction by showing affected migration rates of ctDNA in the presence of these compounds [88]. CD spectra analysis revealed that the addition of CTAT and CNLS reduced the CD signals of ctDNA at 275 nm and caused a bathochromic shift at 245 nm, indicating electrostatic interactions. Furthermore, these promising modified peptides were tested in their destabilization ability against G1 and G2 sequences. The decrease in ellipticity of G1 and G2 CD spectra upon CTAT and CNLS addition emphasized their potentiality in disrupting parallel G4s, with CTAT being more effective [88]. Additionally, the peptide–carbazole conjugates, CTAT and CNLS, significantly increased antibacterial activity up to 4-fold compared to CIBA, NLS, and TAT, indicating their potential as novel antimicrobial agents through the G4-disruption pathway [88].

A G-clamp dimer (Figure 4) was recently revealed to be able to detect two nearby guanines in the stem region of the r(CGG)_n_ repeat and force the G4 structure to transition into a hairpin [89]. The addition of the G-clamp dimer to ThT-r(CGG)_4_ and ThT-r(CGG)_8_ complexes (0.1 µM), in 10 mM HEPES buffer (pH 7.4) containing 100 mM KCl, induced a concentration-dependent drop in fluorescence intensity. ThT fluorescence dropped by roughly 80% with only one equivalent of G-clamp in the instance of r(CGG)_4_ G4 and by about 90% with three equivalents. Otherwise, r(CGG)_8_ G4 showed only a 60% decrease in ThT fluorescence. The CD spectra of r(CGG)_4_ in the K^+^ buffer verified this transition, as the positive peak at 260 nm reduced in ellipticity intensity and shifted to 270 nm with G-clamp addition. The structural conversion from G4 to a hairpin loop structure by G-clamp binding was well validated by RNase T1 assay, highlighting the potential for using this type of ligand to treat disorders linked with RNA G-quadruplex structures [89].

### 2.4. Small Molecules with Tunable G4-Unfolding Properties

**Figure 5:**
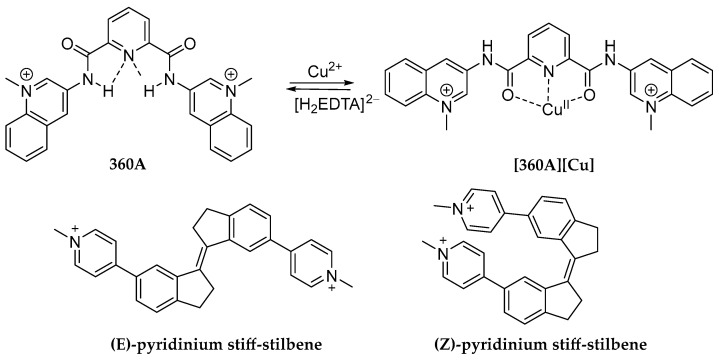
Chemical structures of the ligands that alter their affinity and unfolding capacity in response to G4’s changing experimental circumstances.

External signals can modulate the affinity of a ligand for G4s. This was demonstrated by Monchaud et al., who highlighted that a metal-mediated conformational switch can regulate the G4 binding affinity of a structurally flexible ligand [90]. In this study, the authors demonstrated that Cu(II) could be a G4 switch-off agent. Indeed, when the 360A ligand (Figure 5) was free, it behaved, as is known, as an excellent stabilizing binder for telomeric 22AG G4 (ΔT = 21 °C with 3 µM DNA-G4 in 10 mM sodium cacodylate buffer, 100 mM KCl, 20 °C). Otherwise, by adding high concentrations of Cu(II) (30 equivalents), 360A coordinated the metal-ion, changing its conformations and thus favoring the helix-to-coil transition of 22AG [90]. Indeed, it is known that the addition of high amounts of Cu(II) (>0.7 molar equivalents per nucleotide) can cause nucleic acid structural transitions by the metal-ion interaction with N7 of the guanine residue, skewing the typical hydrogen-bond pairing. Interestingly, the G4 structure may be restored by adding H_2_EDTA^2–^ (30 equivalents). It trapped Cu(II) ions in a complex and favored the release of 360A, which, by returning to its V-shape, strongly stabilized the 22AG G4. The authors also demonstrated that the cyclic of the Cu-mediated structural conversion was reversible with an efficiency of 94% [90]. This study underscored that the internal hydrogen-bond-based molecular organization may be a key parameter for modulating the affinity of a G4-ligand via external stimuli.

Another fascinating example is represented by the two stereoisomers (E and Z) of pyridinium stiff-stilbene proposed by O’Hagan et al. (Figure 5) [91]. A FRET melting assay revealed that both stereoisomers selectively stabilized F21T (5′-FAM-GGGTTAGGGTTAGGGTTAGGG-TAMRA-3′) and FmycT (5′-FAM-TTGAGGGTGGGTAGGGTGGGTAA-TAMRA-3′) G4 structures over duplex DNA (5′-CAATCGGATCGAATTCGATCCGATTG-3′), with (E)-pyridinium stiff-stilbene having the greatest stabilization effect. On the other hand, the CD spectrum of telo23 (4.22 µM, 5′-TAGGGTTAGGGTTAGGGTTAGGG-3′) in the presence of (E)-pyridinium stiff-stilbene indicated no conformational change in 100 mM K^+^ phosphate buffer (pH 7.4) while emphasizing a significant conformational shift in 100 mM Na^+^ phosphate buffer. The addition of 3 to 10 equivalents of the ligand resulted in a hypochromic effect of the positive band at 288 nm, as well as an increase in a positive induced CD signal at about 270 nm. These modifications aided the unfolding of telo23’s hybrid G4 structure in the Na^+^ environment using (E)-pyridinium stiff-stilbene. NMR and metadynamics simulations supported the findings [91]. Fascinatingly, the irradiation of the ligand at 400 nm caused it to lose its ability to unfold G4s. Indeed, when a 10 mM solution of (E)-pyridinium stiff-stilbene in aqueous 100 mM Na^+^ phosphate buffer was exposed to 400 nm light, photo-oxidation, fragmentation, and demethylation processes took place. Nevertheless, the CD spectrum obtained after irradiation was identical to the original spectrum of the G4 in the absence of the ligand, confirming a photo-regulated unfolding capability [91].

## 3. Antisense Strategies

**Figure 6:**
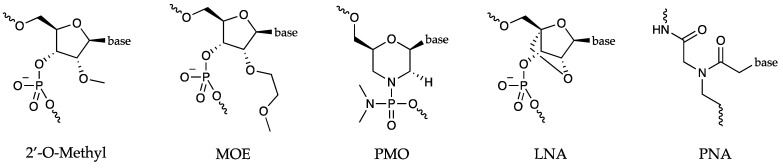
Chemical modifications of various ASOs, 2′-OMe, MOE, PMO, LNA, and PNA.

The antisense strategy in nucleic acid research is a powerful molecular technique used to modulate gene expression. This method employs single-stranded DNA or RNA oligonucleotides that are complementary to specific messenger RNA (mRNA) sequences aiming to interfere with protein translation or mRNA stability. Given the significant transcriptional and post-transcriptional regulation of mRNA and its key role in numerous pathologies, RNA molecules have received particular attention as potential candidates for therapeutic development [92]. Antisense oligonucleotides (ASOs) are single-stranded DNA or synthetic RNA molecules, typically 12–25 nucleotides in length [93]. Their high specificity derives from their ability to complementarily bind to a unique sequence within the total pool of targets present in cells through Watson–Crick base pairs. Upon ASO binding to the mRNA target, a double-stranded hybridization is formed that can interfere with the normal function of the mRNA, causing an inhibition of protein translation, mRNA destabilization, or activation of RNA degradation processes [94]. ASOs are promising therapeutic agents due to their straightforward design, simple conception, relatively low cost, and the ability for chemical modifications to enhance their pharmacokinetic properties, stability, and specificity [93]. However, unmodified ASOs cannot cross the plasma membrane and are highly susceptible to degradation by endonucleases and exonucleases. To overcome these limitations, ASOs have undergone chemical modifications [95], with the most promising results in locked nucleic acids, phosphorodiamidate morpholino oligomers, and peptide nucleic acids (Figure 6). These tools have enhanced stability and reduced toxicity, forming a more stable hybrid with their target [94]. In addition, ASOs feature alkyl modifications at the 2′-position of the ribose, such as oxygenated groups leading to the formation of 2′-O-methyl (2′-OMe) and 2′-O-methoxyethyl (2′-MOE) moieties (Figure 6), exhibit a slightly higher affinity for their target than traditional ASOs, and are incompatible with recruitment and cleavage by RNase H [96]. ASOs are significant in pharmacological research where they are used to study gene function and their protein products, as well as in gene therapy, in which they are employed to modify gene expression to treat hereditary illnesses [97]. Among all, they are employed to modulate G4-folding, affecting mRNA translation. For instance, ASOs can interfere with G4 formation in critical regions of oncogenes, thereby reducing their expression and tumor proliferation. Conversely, they can be employed to promote G4 folding in regions of genes that play a protective role, enhancing their expression and counteracting disease progression [98,99]. Therefore, the antisense approach offers a new perspective in gene therapy and biomedical research, enabling a precise modulation of G4 conformation for therapeutic purposes.

### 3.1. 2′-OMe Antisense Oligonucleotides in mRNA-G4 Unfolding

**Figure 7:**
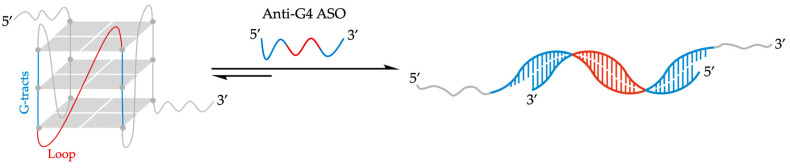
Schematic representation of the oligonucleotide-based strategy to disrupt G-quadruplex structures. The best-performing strategy lies in hybridizing the central loop and the two adjacent G-tracts on both sides. Blue lines represent G-tracts; red lines represent the central loop in the G4 structure. The complementary bases in the Anti-G4 ASO were represented with the same colors.

It was hypothesized that ASO sequences designed to hybridize with a G4 loop together with both the adjacent G-tracts would be highly effective in preventing or disrupting G4 folding (Figure 7). Rouleau et al. exploited an artificial RNA-G4 with an extended 13-mer loop (Art-G4) to emphasize the power of this approach [98]. They synthesized 2′-OMe-RNA ASO sequences complementary to the central loop and the two adjacent G-tracts to ensure high-affinity binding and heteroduplex stability. In-line probing confirmed that a 17-mer Anti-G4 ASO (5′-CCCAACGUCGCUGCAACCC-3′) could hinder Art-G4 formation, even in the presence of 100 mM KCl in 50 mM TRIS-HCl buffer, pH 7.5. Unfortunately, this system showed only limited unwinding effects on pre-folded Art-G4 under the same experimental conditions [98]. When Art-G4 was transfected into HEK293 cells using a luciferase reporter gene, the addition of Anti-G4 ASO led to enhanced Art-G4 expression. This suggested that ASO binding interfered with G4-folding, thus increasing mRNA production [98]. The inhibitory effect on G4 folding was directly proportional to the length of Anti-G4 ASO, with the 19-mer (complementary to the 13-mer central loop and the 3 + 3 adjacent guanines) showing the best results with 47% inhibition of G4-folding [98]. Interestingly, a mismatch with three loop bases was enough for non-significantly affected Art-G4 formation. The potentiality of this approach was further tested on H2AFY mRNA to demonstrate its applicability to naturally occurring G4s. The 5′-UTR of mRNA isoforms 1, 3, and 4 of the H2AFY gene contains a G4 with a 13-mer loop that was targeted by a specific anti-H2G4 ASO showing a G4-folding inhibition by in-line probing experiment. Luciferase constructs containing the H2AFY 5′-UTR were transfected into HEK293 cells, showing a reduction in mRNA translation in the presence of G4 and a further reduction in the presence of the anti-H2G4 ASO. However, the effect of the G4 structure appears to be limited in cells due to potential competing secondary structures in the 5′-UTR. To demonstrate the versatility of the ASO approach, another G4 located in the 5′-UTR of the Akirin2 mRNA was considered. Experiments show that the anti-AkG4 ASO does not significantly increase luciferase translation, suggesting that G4 folding was not inhibited. However, the use of a DNA/LNA hybrid with a phosphorothioate backbone ASO instead of a 2′-OMe ASO reduced the G4 folding [98]. Subsequently, it was demonstrated that ASOs could also influence G4 folding in endogenous H2AFY mRNA in Caco-2 cells, a cellular model for colorectal cancer. The anti-H2G4 ASO increased H2AFY expression, while the pro-H2G4 ASO reduced it, confirming the efficacy of ASOs in targeting endogenous G4 structures. In conclusion, this study highlights that ASOs can be utilized to modulate the folding of G4 structures in mRNAs, thereby influencing gene expression. These findings have significant implications for gene regulation and the development of novel therapies based on RNA structure manipulation [98].

### 3.2. Effective Peptide Nucleic Acid Sequences

Third-generation ASOs, characterized by chemical modifications of the furanosic nucleotide ring, were developed to further improve nuclease resistance and target affinity, biostability, and pharmacokinetic properties. Among these, peptide nucleic acids (PNAs) introduced by Nielsen in 1991, are analogs of DNA/RNA, in which the sugar-phosphate backbone is replaced by a pseudo-peptide (N-(2-aminoethyl)glycine) [100]. This structure allows natural nucleobases to attach via methylene carbonyl bonds, enabling binding to complementary DNA/RNA through Watson–Crick base pairing [101]. PNAs exhibit high binding affinity and hybridize with high specificity due to the absence of electrostatic repulsion. While PNA/DNA hybrids can be oriented in parallel or antiparallel modes, the antiparallel mode is more stable due to PNA’s achiral backbone [102]. These hybrids are highly selective, surpassing DNA probes in recognizing single-base mismatches [103]. The peptidyl backbone also grants PNAs resistance to nucleases and proteases, providing stability across a wide pH range [104]. The greater thermodynamic and enzymatic stability and affinity of oligonucleotide analogs explain why they are used in preference to normal ones. However, the large hydrophilic PNA molecules face challenges in cellular uptake, limiting their experimental use in gene regulation. This limitation can be overcome by techniques like microinjection, electroporation, and co-transfection with DNA, or by conjugating PNAs with negatively charged oligomers, lipophilic companies, or peptides that penetrate cells. Such modifications can also improve solubility and reduce aggregation without compromising binding properties, and can even introduce chirality, charge, or additional functionality [105]. Based on the unique properties, several approaches have been developed to modulate G4 folding with PNAs.

**Figure 8:**
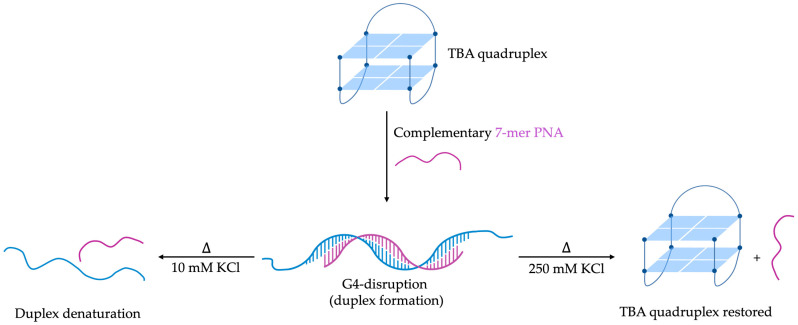
Schematic representation of invasion of TBA quadruplex by complementary 7-mer PNA (P1), and the effect of ionic strength on thermal denaturation pathway of TBA-P1 hetero duplex.

An interesting example of G4 disruption by PNA sequences was reported by Datta et al. in 2001 [106]. They demonstrated the efficacy of a 7-mer PNA sequence (P1) in unfolding the fifteen-base TBA, known for its stable antiparallel intramolecular G4 structure composed of two stacked guanine tetrads and three loops [107]. P1 (H-CCACACC-Lys-NH_2_) was specifically designed to complementarily hybridize with the seven central nucleobases of the TBA sequence, targeting critical regions for G4 stability. The formation of a stable PNA-DNA duplex was confirmed by the CD spectrum of the 1:1 mixture of P1 and TBA (5 μM each, in 10 mM KCl, 10 mM KPi buffer, pH 7.2), featuring a maximum at 262 nm and a minimum at 238 nm [106]. Specifically, this spectrum is typical of PNA-DNA duplexes in which the N-terminus of PNA aligns with the 3′-terminus of DNA, consistent with the antiparallel orientation favored by PNA sequences. Under these experimental conditions, the newly formed P1-TBA heteroduplex exhibited remarkably high thermal and thermodynamic stability, with a T_m_ of 55.2 ± 0.2 °C and a ΔG at 298 K of −11.7 ± 0.1 kcal/mol (as determined by UV melting assay) [106]. Significantly, the P1-TBA hybrid denatures at a temperature 8.5 °C higher than the G-quadruplex TBA (T_m_ = 46.7 ± 0.2 °C by CD melting assay), preventing the DNA strand from refolding into G4 structure upon P1-TBA dissociation (Figure 8). The stability of the duplexes formed during PNA hybridization was observed to increase in the presence of overhangs from unpaired DNA. This phenomenon, termed “overhang stabilization”, is attributed to the additional bases stacking onto the duplex, thereby enhancing the stability of the complex. However, increasing the ionic strength to 250 mM KCl concentration (twice the physiological environment KCl concentration) [108], led to a drastic reduction in the melting temperature of P1-TBA heteroduplex (T_m_ = 34 ± 2 °C; ΔG_298 K_ = −8.3 ± 0.1 kcal/mol by UV melting assay), favoring the folding of TBA into a G4 structure once the PNA-DNA hybrid denatured. Nevertheless, the authors suggested that the decrease of 3.4 kcal/mol in the driving force was not attributable to a decrease in hybrid stability but rather to an increase in the stability of TBA [106].

**Figure 9:**
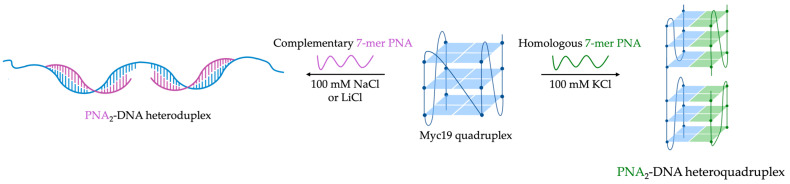
Schematic representation of PNA_2_-DNA heteroduplex and heteroquadruplex formed by Myc19 DNA quadruplex and complementary or homologous 7-mer PNA.

The same research team compared the impact of hybridization using either homologous (G-rich sequences identical to the target, promoting intermolecular G4 formation) or complementary (C-rich sequences targeting the G4 to form a duplex) 7-mer PNAs [109]. Their findings revealed that different G4 conformations exhibit variable responses to homologous and complementary probes, influenced by ionic conditions [110]. Two topologically different G4s were, therefore, investigated by surface plasmon resonance (SPR) experiments, CD, and heteroduplex formation studies using cyanine dyes. In particular, Myc19 DNA-G4 (sequence modeling the promoter region of the MYC proto-oncogene; 5′-AGGGGGGGGGGGGGA-3′) adopts a parallel morphology with anti-oriented guanines. In high K^+^ concentration (100 mM), Myc19 readily forms intermolecular hetero-quadruplex (PNA_2_:DNA) with a homologous probe (P_myc_H: H-GGGGAGG-LysNH_2_) (Figure 9). However, disruption with the complementary probe (P_myc_C: H-CCCCACCC-LysNH_2_) was less effective. Only by weakening the DNA-G4 through the substitution of potassium with sodium or lithium can the complementary PNA bind effectively to the Myc19 target. Conversely, in a K^+^-rich buffer (100 mM), the hybrid G4 formed by h-Telo22 (quadruplex modeled on the human telomeric sequence; 5′-AGGGTTAGGGTTAGGG-3′), did not rearrange into an intermolecular hetero-quadruplex with a homologous probe (P_telo_H: H-GGGTTAGGG-LysNH_2_). Instead, it promptly forms a PNA_2_:DNA heteroduplex with a complementary probe (P_telo_C: H-CCCTAACCC-LysNH_2_), leading to the G4 unfolding. Furthermore, the formation of the heteroduplex is favored under all ionic conditions [109].

**Figure 10:**
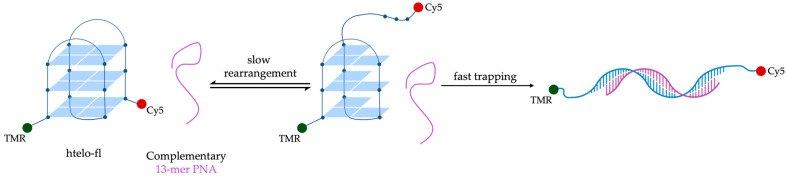
Schematic representation of the mechanism htelo-fl unfolding with complementary 13-mer PNA.

Green et al. demonstrated the efficient disruption of the 21-mer human telomeric G4 sequence, 5′-d(GGGTTAGGGTTAGGG)-3′ (htelo) by a 13-mer complementary sequence [111]. Fluorescence spectra (100 mM NaCl and 10 mM Tris-HCl, pH 7.4) of a dual-labeled htelo (htel-fl) with tetramethylrhodamine and Cy5 at the 5′ and 3′ ends, respectively, revealed that while the non-complementary PNA (nPNA: H_2_N-TGTAAGGAACTAG-Lys-CO_2_H) had minimal effects on the fluorescence spectrum, the addition of the complementary PNA (cPNA: H_2_N-CTAACCCTAACCC-Lys-CO_2_H) significantly reduced Cy5 fluorescence. This behavior indicated the opening of htelo-fl sequence with the formation of a PNA:DNA duplex (Figure 10) [111]. FRET experiments revealed first-order kinetics, suggesting that the G4 undergoes a rearrangement before being trapped by cPNA. The unfolding process was independent of PNA concentration, indicating that the rate-determining step is the internal rearrangement of the G4, followed by rapid hybridization with the cPNA. However, the structural details of this rearrangement could not be determined, and it remains possible that htelo-fl must fully unfold to allow for cPNA hybridization. An Arrhenius analysis of the system yielded an activation energy of 98 ± 8 kJmol^−1^, comparable to values found for other quadruplexes [111]. This study highlights the potential of using PNA traps to probe the dynamics of G4 structures and underscores the complexity of their unfolding mechanisms.

**Figure 11:**
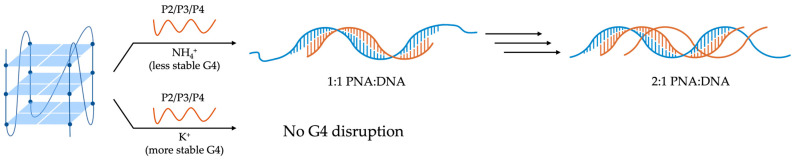
Schematic representation of the comparison of the cations’ effects (NH_4_^+^ versus K^+^) on the unfolding of cKit87up quadruplex in the presence of P2–P4 PNAs.

In 2011, the group of Oliviero investigated a small library of PNAs designed to target the cKit87up G4 structure in solutions containing either potassium or ammonium ions [112]. PAGE experiment preliminarily demonstrated that comparable folding patterns were achieved by cKit87up G4 (10 μM) both in 50 mM KCl or 150 mM NH_4_OAc solutions at pH 7.0. CD spectroscopy further confirmed the formation of parallel G4s in both conditions, while UV melting studies revealed differences in thermal stability between the potassium and ammonium solutions, with cKit87up G4 showing higher stability in potassium (T_1/2_ = 47.3 °C) compared to ammonium (T_1/2_ = 34.3 °C). Thermal difference spectra collected under both conditions exhibited characteristic patterns of a G-quadruplex structure [112]. To verify potential cKit87up G4-disruption, four different PNA sequences (P1–P4) targeting different stretches of the ckit87up sequence were examined. The 5-mer P1 (H_2_NLys-TCCTC-H) was fully complementary to the five bases of the long loop (A16-G20), while P2 (H_2_NLys-TCCTCCC-H) and P3 (H_2_Lys-TCCCTCCC-H) were complementary to the last seven and first eight nucleosides of the sequence, respectively. A longer PNA was also selected P4 (H_2_NLys-CCCTCCCGCGAC-H), which is fully complementary to the first thirteen nucleosides of cKit87up. P1 was found to preserve the G4 structure in both potassium and ammonium solutions, as evidenced by nearly identical CD profiles to the one of cKit87up G4 alone in potassium buffer [112]. By contrast, PNAs P2–P4, which targeted different segments of the cKit87up sequence, showed distinct binding behaviors depending on the cation present. In an ammonium solution, P2–P4 acted as disruptive agents, unfolding the G4 structure and forming stable PNA/DNA hybrid complexes (Figure 11), as observed through shifts in CD spectra and changes in PAGE mobility. ITC data indicated two binding events for P2–P4, with the formation of 1:1 and 2:1 PNA/DNA complexes. These interactions were enthalpy-driven, suggesting stable binding and disruption of the quadruplex structure [112]. Since it is known that pyrimidine-rich PNA sequences can invade DNA strands by forming PNA-DNA-PNA triplexes, the authors believe that PNA:DNA 2:1 complexes, observed in ammonium in the presence of an excess of P2, P3, and P4, may be PNA-DNA-PNA-triplex-type complexes (Figure 11). Conversely, in a potassium solution where the cKit87up G4 was more stable, P2–P4 were able to bind to the G4 without unfolding it, maintaining its structural integrity as confirmed by CD, UV, and PAGE experiments (Figure 11). The findings underscored the significant influence of cation type on the stability and interactions of G4 structures with PNAs. In the NH_4_^+^ solution, PNAs can overcome the less-stable cKit87up G4 structure by hybridizing the DNA sequence. By contrast, in the K^+^ solution, where the stability of cKit87up G4 is higher, the PNAs are still able to bind to the G4 without overcoming it [112].

Very recently, to disrupt the highly stable G4 structure within the transcription start site of the SNCA gene, a short PNA sequence was synthesized [34]. This PNA was designed to hybridize with the central bases of the SNCA G4 (from G10 to G18), facilitating the formation of a PNA-DNA duplex and unfolding the G4 structures. To enhance nuclear delivery, a nuclear localization sequence, a lysine residue, and a Coumarin 343 (C343) dye were conjugated to the PNA, improving water solubility and cellular uptake (H_2_NCO-NLS-Gly-CCCCGTCCC-Lys-C343). pSNCAext unfolding was indicated by a sharp shift in the dichroic signal, with a positive band at 278 nm and a negative band at 252 nm, which indicated the development of a PNA-DNA duplex. The resulting heteroduplex had a melting temperature of 54.3 ± 0.5 °C, demonstrating stable interaction at physiological temperature. Principal component analysis of the CD spectrum data further confirmed the distinct structural characteristics of the new duplex, differentiating it from typical G4 structures [34].

To improve affinity versus the complementary nucleic acid target, PNA oligomers with a substituent at the γ-position, called γPNA, have been proposed. This substitution induces a pre-organization of the entire sequence into a helix by enhancing binding potency. In this context, Oyaghire et al. studied the position-dependent effects of γPNA complementary oligomers for reaching an effective RNA-G4 disruption of 4G3, a sequence within the 5′-UTR mRNA of the NRAS gene [113]. They designed three different 12-mer γPNAs: γ5′ targeting five nucleotides of the 5′-flanking region and the adjacent two G-tracts; γCen binding the central G-tracts; and γ3′ complementary to the last G-tract and the subsequent five flanking bases at 3′-end. Among these, γ5′ showed a more potent dose–response inhibition of the luciferase mRNA translation than other γPNAs, especially at lower concentrations (ranging from 10 to 90 nM; RNA concentration equal 10 nM), with an IC_50_ value 5–6 folds lower (15 nM at 37 °C). This higher efficiency was not directly dependent on different affinities, considering that the Kd values varied by a factor of only two (4.2 nM for γ5′ by SPR), but on kinetic preference in targeting 5′-end of 4G3. γ5′ resulted to be more potent than 2′-OMe oligomer (around 15-fold) and more selective than PNA5′, which caused a higher non-specific decrease in luciferase mRNA translation of no G4 control [113].

### 3.3. Locked Nucleic Acids as One of the Latest Advancements in Nucleotide Analogs

Firstly proposed in 1998, Locked Nucleic Acids (LNAs) are modified oligonucleotides utilized in both biotechnological and therapeutic applications due to their exceptional stability and ability to enhance the affinity and specificity toward complementary DNA and RNA [114]. Characterized by a methylene bridge linking the 2′ oxygen to the 4′ carbon of the furanose sugar [115], LNAs effectively “lock” the flexibility of the phosphodiester bond, maintaining the ribose group rigidly in the C3′-endo/N-type conformation. This unique bicyclic structure plays a pivotal role in the remarkable binding ability of LNA nucleosides, serving as a scaffold for other bicyclic analogs with similar features [116]. LNAs are primarily categorized into two types: mixmers and gapmers. Mixmers consist of a random arrangement of LNA and DNA nucleotides, while gapmers feature two LNA multinucleotide fragments flanking a central DNA portion. Both types can hybridize with DNA or RNA filaments or be conjugated with other molecules [117]. LNAs offer several advantages over conventional oligonucleotides, including increased thermal stability of duplex helices with DNA or RNA, leading to better molecular recognition and binding. Indeed, LNA/DNA or LNA/RNA hybrids exhibit higher melting temperatures compared to DNA/DNA hybrids due to enhanced base-stacking interactions and backbone preorganization [118]. They are highly compatible with other monomers and can be seamlessly integrated with various types of ASOs or natural DNA and RNA [119]. Leveraging their high specificity and stability, LNAs hold potential for novel treatments of genetic, oncological, and viral diseases [119] and, therefore, are excellent candidates for developing probes to disrupt the G-quadruplex structures.

**Figure 12:**
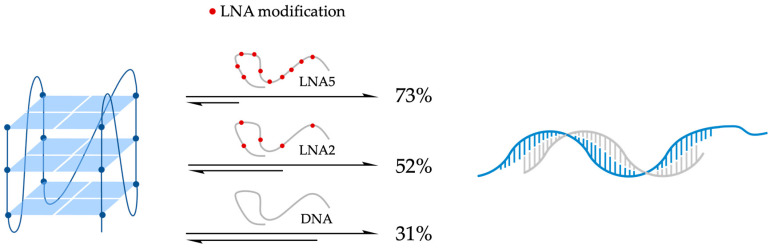
Schematic representation of the effect of the number of LNA modifications (LNA5 10 modifications, LNA2 5 modification, and control DNA) on the unfolding of a G-quadruplex structure formed by a 22-mer purine-rich sequence of NHE III of the c-MYC. The percentage values represent the amount of duplex formation, consistently with G4-unfolding.

The P1 promoter of the c-MYC gene contains a nuclease hypersensitive element (NHE) with a quadruplex structure, crucial for its biological function [120]. Maiti and colleagues investigated the balance between this G4 and the conventional Watson–Crick duplex using LNA-modified complementary strands. They targeted a purine-rich 22-mer sequence (5′-GGGGAGGGTGGGGAGGGTGGGG-3′) from the NHE III of the c-MYC promoter, located from −143 to −110 bp upstream of the P1 promoter [121]. Through NMR and CD techniques, they identified multiple interconverted conformers and the formation of a stable quadruplex with a parallel component. They then explored the invasion of this quadruplex using a library of five systematically modified LNA-rich complementary strands. LNA monomers were incorporated complementary to the guanine bases in the G-tetrads, loop bases, and diagonal regions of the quadruplex. FRET experiments showed that the addition of LNA-modified strands to a pre-folded G4 (1:1 ratio, 100 nM, in 10 mM sodium cacodylate buffer, 140 mM KCl, and 100 mM NaCl at pH 7.4) increased duplex formation compared to unmodified strands [121]. Specifically, LNA 5, with ten modifications (5′-CCCCACCCTCCCCACCCTCCCC-3′) showed the highest duplex formation efficiency at a 1:1 ratio, reaching 73% (Figure 12). Under the same conditions, LNA-2 (5′-CCCCACCCTCCCCACCCTCCCC-3′), with only five LNA alterations induced 52% duplex formation, whereas the unmodified DNA strand produced only 31% duplex formation (Figure 12). A full duplex creation required a 15-fold excess of the original DNA strand. Non-denaturing gel electrophoresis (1.5 μM G4, in the absence and presence of equimolar concentrations of unmodified and modified complementary strands in 100 mM NaCl buffer at pH 7.4 and 4 °C) better confirmed the G4 conversion into a duplex with LNA-modified strands. The extent of this conversion was dictated by the number of introduced modifications, with the best results achieved with LNA 5, containing the highest number of modifications, which showed nearly complete conversion into hetero LNA-DNA duplex [121]. Besides the number of modified bases, their position is also crucial for superior performance. Surface plasmon resonance studies, used to obtain kinetic parameters involved in the hybridization process, demonstrated an increase in the association rate (Ka) and a decrease in the dissociation rate (Kd) with more LNA modifications. Fascinating, LNA 5 exhibited a binding affinity one order of magnitude higher (Ka = 4.6 × 10^5^ M^−1^ s^−1^) than unmodified DNA (Ka = 1.5 × 10^5^ M^−1^ s^−1^) with concomitant 3-fold lower dissociation rate (Kd = 0.31 × 10^−3^ s^−1^ for LNA 5 and Kd = 1.0 × 10^−3^ s^−1^ for unmodified DNA). In potassium buffer, which significantly stabilizes the G4 (T_m_ = 75 °C), LNA-modified strands still outperformed unmodified strands in driving duplex formation. A complete G4-LNA duplex formation was achieved with a 10-fold excess of the LNA complementary strand in 140 mM KCl solution [121]. In vivo experiments in zebrafish, using a plasmid with the target sequence upstream of a luciferase reporter gene, showed that LNA-modified strands significantly suppressed luciferase expression compared to unmodified strands. This suppression was proportional to the number of LNA modifications (DNA < LNA 2 < LNA 5), indicating that LNA-modified strands facilitate the transition to duplex effectively disrupting G4-folding, leading to interfering with molecular recognition and reducing gene expression. Overall, the study demonstrates that LNAs can efficiently target and disrupt G4 structures, shifting the balance towards duplex formation and potentially downregulating gene expression. This strategy of using LNA-modified oligonucleotides to exploit the G4-duplex structural switch may lead to more effective and selective therapies for specific human tumors [121].

**Figure 13:**
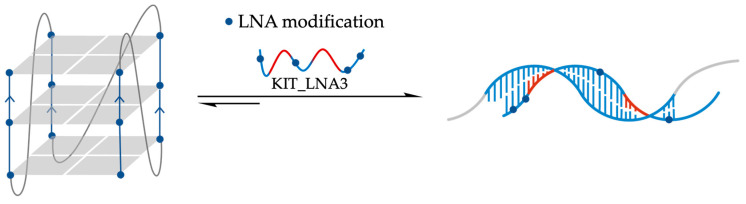
Schematic representation of the unfolding of cKIT1 G4 by KIT_LNA3 by targeting the central guanine of each G-tract. The red tract is related to loop bases.

Very recently, Chowdhury et al. investigated the position-dependent effect of LNA modifications on the unfolding of the c-KIT1 G4 [122]. Specifically, five oligonucleotides were synthesized, each with four LNA modifications at different positions within the c-KIT sequence: KIT_LNA1 (5′-CCCTCCTCCCAGCGCCCTCCC-3′) targeted the 3′ loop; KIT_LNA2 (5′-TCCCTCCTCCCAGCGCCCTCCC-3′) focused on the 3′ G-tract; KIT_LNA3 (5′-TCCCTCCTCCCAGCGCCCTCCC-3′) targeted the central guanine of each G-tract; and KIT_LNA4 (5′-TCCCTCCTCCCAGCGCCCTCCC-3′) contained random LNA modifications along the loops of the c-KIT1 G4 sequence. UV melting experiments assessed the stability of the DNA-LNA heteroduplexes formed between c-KIT1 and the complementary LNA probes. All LNA probes caused a significant increase in the melting temperature of the formed duplex (~8 °C) compared to the duplex formed using an unmodified DNA probe (KIT_DNA1, 5′-TCCCTCCTCCCAGCGCCCTCCC-3′). This indicates that the positioning of LNA modifications has a limited effect on the thermodynamic stability of the duplex post-G4 disruption [122]. By FRET-based assay, the authors highlighted a position-dependent effect of LNA modification on the G4 disruption kinetics. Specifically, invasion half-time of c-KIT G4 treated with the unmodified probe KIT_DNA1 (100 nM of labeled c-KIT1 annealed in the presence of 5 equivalents of KIT_DNA1 probe, in a Tris-HCl buffer, pH 7.4, containing 1 mM KCl) was 1753 ± 70 s. Otherwise, LNA-modified probes showed significant variations in G4 disruption rates depending on the LNA monomers positioning. KIT_LNA3, with modifications opposite the G tetrads, significantly decreased the G4 disruption half-life to 628 ± 40 s, accelerating the process by ~3 times (Figure 13). KIT_LNA1, targeting the loops, had a half-life comparable to KIT_DNA1 (1481 ± 144 s). KIT_LNA2, targeting the 3′ G tetrad, also reduced the half-life (1087 ± 24 s), while KIT_LNA4, with random modifications, increased the half-life (4247 ± 494 s). In summary, LNA modifications, especially when positioned at the guanines involved in G-tetrads, can significantly accelerate c-KIT1 G4 unfolding, indicating a novel potential strategy for designing effective G4-disrupting probes [122]. The researchers extended their study to the human hTelo sequence, which adopts a mixed-type topology. They designed LNA-modified probes targeting the G-tetrads (hTelo_LNA1, 5′-CCCTAACCCTAACCCTAACCC-3′) and with random modifications (hTelo_LNA2, 5′-CCCTAACCCTAACCCTAACCC-3′). The hTelo_LNA1 significantly reduced the G4 disruption half-life (1539 ± 18 s), while hTelo_LNA2 increased the half-life (2179 ± 39 s), confirming the position-dependent effect of LNA modifications on G4 unfolding. Additionally, FRET disruption assays (100 nM of G4 in Tris-HCl buffer at pH 7.4 with 1 mM K^+^) demonstrated that short probes, such as KIT_LNA_short (5′-TCCCTCCTCC-3′) for c-KIT1 and hTelo_LNA_short (5′-CCCTAACCCT-3′) for hTelo, significantly accelerated G4 disruption more efficiently than longer LNA-modified versions (KIT_LNA_2 and hTelo_LNA1). Short unmodified DNA probes (KIT_DNA_short) did not disrupt the G4, underscoring the critical role of LNA modifications [122]. To verify these results under physiological conditions, PAGE gel analyses were conducted with c-KIT1 in high K^+^ concentrations (25 nM of cKIT1 labeled with Cy5, in Tris-HCl buffer at pH 7.4 containing 100 mM KCl with a 10-fold excess of the probe), highlighting that only KIT_LNA_short formed an LNA-DNA heteroduplex, while KIT_DNA_short did not [122]. To break down hTelo G4s, short LNA probes have been effectively used in a single-molecule optical tweezer device. This finding implies that stable G4s, which could normally prevent polymerase from functioning properly during transcription or replication, can be broken by LNA probes. A single-step extension assay was used to evaluate the potential use of such LNA probes in unwinding G4s in biochemical processes [123]. While their equivalent short DNA iso-sequences did not display the same efficiency, it was shown that short LNA probes can disrupt G4s and increase polymerase extension. A dual luciferase test [124] was then carried out to evaluate how the probes affected cellular transcription. Short LNA probes aimed at c-KIT1 G4 were found to be able to raise gene expression in cells using a plasmid system containing the c-KIT oncogene promoter region. Comparable DNA sequences, however, had a different outcome. These findings provide credence to the idea that LNA probes can prevent cells from going through G4, creating new opportunities for the development of LNA-based selective G4 disruptors and the investigation of the cellular biology of these DNA structures [122].

## 4. Conclusions

In this review, we summarized key advances in G4 unfolding, a field less explored compared to G4 stabilization. We highlighted the potential of exploiting small molecules and antisense strategies to selectively disrupt G4 structures, paving the way for innovative therapeutic applications and a deeper understanding of G4 roles in biological processes.

The use of small molecules to disrupt G-quadruplex structures holds significant promise for therapeutic development. Their specificity, therapeutic versatility, ease of administration, and potential to provide mechanistic insights make them powerful tools in the fight against various diseases. As research in this field advances, the continued development and optimization of G4-targeting small molecules will likely lead to innovative treatments that exploit the unique properties of G4s. Therefore, the need for establishing consistent and reliable methods for defying effective destabilization effects is essential to facilitate more accurate comparisons between studies, ultimately leading to more effective and specific G4-targeting therapeutic agents. Here, starting with TMPyP4, which provided the first evidence of G4 unfolding activity, we reported a wide variety of small molecules that demonstrated their ability to unfold DNA and RNA G4 structures. Along with destabilizing G4-ligand, we also reported well-known stabilizing ligands that can unfold the G4 structures upon external stimuli.

At the same time, antisense strategies are increasingly emerging as a highly effective alternative in gene therapy. This technique uses antisense oligonucleotides to modulate gene expression with high specificity, providing promising therapeutic approaches also by exploiting G4 disruption. Specifically, we reported 2′-OMe, PNA, and LNA systems that demonstrated encouraging prospective in terms of G4 destabilization efficiency. PNAs are favored for their thermodynamic, enzymatic stability, and target affinity. LNAs, instead, exhibit superior thermal stability, molecular recognition, and binding due to enhanced base-stacking interactions and backbone preorganization. In general, their complementarity with guanine bases in G-tetrads makes them significantly more efficient G4-unfolding agents both in vitro and in vivo.

Looking ahead, combining small molecules and antisense strategies could harness the best features of both approaches to achieve selective and efficient disruption of G4 structures. This could pave the way for innovative therapeutic applications in a disparate number of diseases. Continued focus on this emerging research field is essential to better understand the key role of G4s and their involvement in biological processes.
